# Advances in Intelligent Vehicle Control

**DOI:** 10.3390/s22228622

**Published:** 2022-11-09

**Authors:** Juan A. Cabrera

**Affiliations:** Department of Mechanical Engineering, University of Malaga, 29071 Malaga, Spain; jcabrera@uma.es

Advanced intelligent vehicle control systems have evolved in the last few decades thanks to the use of artificial-intelligence-based techniques, the appearance of new sensors, and the development of technology necessary for their implementation. Therefore, a substantial improvement in vehicle safety, comfort, and performance has been achieved. The appearance of new vehicles with new technologies incorporated in them requires new control strategies that will continue to increase handling, stability, and energy efficiency.

In recent years, intelligent vehicle control has been widely investigated from different points of view. Many researchers have studied active safety systems, advanced driver assistance systems, autonomous driver systems, etc., through strategies incorporating aspects of artificial intelligence, making them adapt and learn from situations never explored before. To achieve this, it has been necessary to develop increasingly precise dynamic vehicle models and incorporate new intelligent sensors and sensor fusion techniques to learn the vehicle’s state accurately. However, it is important to observe not only the state of the vehicle where these systems are incorporated but also those of vehicles around it that can influence the vehicle’s behavior. This requires communication between vehicles and developing architectures that enable smart transportation. On the other hand, the incorporation of electric vehicles (EVs) in recent years has enabled a new way of focusing on vehicle control systems, fundamentally due to the incorporation of new systems that must be studied differently.

Today, there are still many challenges in this field of research that have to be solved or improved, and that is why the Special Issue, “Advances in Intelligent Vehicle Control” in the journal *Sensors*, has compiled 11 works that have tried to provide an answer to the initial issues raised in it, such as:

- Development of intelligent control algorithms for active safety systems [1,2].
- Smart sensors: development of advanced strategies using future smart sensor technology and intelligent sensor fusion for the measurement and estimation of vehicle states, tire and road conditions, situation awareness assessment, environment mapping, fault diagnosis, and driving conditions [3,4,5,6].
- Intelligent and efficient driving: advanced vehicle control systems for assisted and autonomous driving and vehicle navigation by incorporating new sensors and measurement systems to develop new strategies to avoid critical driving situations and save energy [7,8,9,10,11].

The article titled “Investigation on the Model-Based Control Performance in Vehicle Safety Critical Scenarios with Varying Tyre Limits” [1] was intended to investigate the possibility of physical model-based control to consider the variations in terms of the dynamic behavior of the systems and of boundary conditions. Different scenarios with specific tire thermal and wear conditions were tested on diverse road surfaces, validating the designed model predictive control algorithm in a hardware-in-the-loop real-time environment and demonstrating the augmented reliability of an advanced virtual driver aware of available information concerning the dynamic limits of the tire.

In the article “Nonlinear Ride Height Control of Active Air Suspension System with Output Constraints and Time-Varying Disturbances” [2], addressed the problem of nonlinear height tracking control of an automobile active air suspension with output state constraints and time-varying disturbances. The proposed control strategy guaranteed that the ride height stayed within a predefined range and converged close to an arbitrarily small neighborhood of the desired height, ensuring uniform ultimate boundedness. The authors designed a nonlinear observer to compensate for time-varying disturbances caused by external random road excitations and perturbations, achieving robust performance. Co-simulation showed the efficiency of the proposed control methodology.

The article, titled “Roll Angle Estimation of a Motorcycle through Inertial Measurements” [3], deals with a method to estimate the roll angle in a motorcycle. They developed a multibody motorcycle model and used an observer based on a Kalman filter to estimate the roll angle. The multibody model is a seven-element assembly without closed kinematic loops, where six elements belong to motorcycle parts, and one of them represents the torso of the driver. This model used 12 Degrees of Freedom (DOF). Six DOF from the chassis rigid body condition; five revolute joints from the two wheels, swingarm, steer, and torso roll movement; and one prismatic joint between the fork bars and fork bottles. One of the most important parts of this model is the tire behavior and properties. The authors used a toroidal tire defined with an outer radius, which represents the undeformed outer radius of the tire, and the torus tube radius, which should be selected to represent the tire curvature near the contact patch in the most accurate way. To test their roll angle estimation algorithm, they performed maneuvers in six different scenarios. From these maneuvers, some measurements were obtained, mimicking the properties of actual sensors by adding some white Gaussian noise. These measurements were used to verify the performance of the state observer.

In the article “Semantics Aware Dynamic SLAM Based on 3D MODT” [4], the authors proposed a framework to solve the dynamics of Simultaneous Localization and Mapping (SLAM) problems. They used a Visual-LIDAR based on Multiple Object Detection and Tracking (MODT) to handle the dynamic regions of the scene. The framework was tested on a dataset developed for LIDAR-based autonomous driving and evaluated and contrasted with state-of-the-art SLAM algorithms. The results suggest that the proposed dynamic SLAM framework can perform in real time with budgeted computational resources. In addition, the fused MODT provides rich semantic information that can easily be integrated into SLAM.

The article, titled “A Redundant Configuration of Four Low-Cost GNSS-RTK Receivers for Reliable Estimation of Vehicular Position and Posture” [5], proposed a low-cost sensor system composed of four GNSS-RTK receivers to obtain accurate position and posture estimations for a vehicle in real time. The four receiver antennas are positioned so that each combination of three antennas is optimal for obtaining the most accurate 3D coordinates with respect to a global reference system. The redundancy provided by the fourth receiver allows further improvement of the estimates and maintains accuracy when one of the receivers fails. They carried out successful experiments with a ground rover on irregular terrain. Angular estimates similar to those of a high-performance IMU were achieved in dynamic tests.

In [6] “Deep Transfer Learning Based Intrusion Detection System for Electric Vehicular Networks”, the authors proposed a deep-transfer-learning-based Intrusion Detection System (IDS) model for an In-Vehicle Network (IVN) along with improved performance compared to several other existing models. The unique contributions included effective attribute selection, which is best suited to identify malicious CAN messages and accurately detect normal and abnormal activities, designing a deep-transfer-learning-based model and evaluating capacity considering real-world data. To this end, an extensive experimental performance evaluation was conducted. The architecture, along with empirical analyses, showed that the proposed IDS greatly improves detection accuracy over mainstream machine learning, deep learning, and benchmark deep transfer learning models and demonstrated better performance for real-time IVN security.

The articles, titled “Service-Centric Heterogeneous Vehicular Network Modeling for Connected Traffic Environments” [7], deals with connected vehicles using mobile networks. In this article, a heterogeneous network model for heterogeneous vehicular communications is presented. After developing the network model, the following conclusions were reached:

- Network cooperation supports cloud computing on big traffic data to realize intelligent traffic services.
- A heterogeneous network coordinator and gateway are the key to proper connection management.
- Service-oriented traffic applications become smarter with increased traffic data mastery and processing power.
- Practical simulation verified higher message diversion and flow utilization and a lower rate of message loss and delays for traffic services when implementing heterogeneous vehicular communications.
- The mathematical modeling of a service-oriented network prioritization and implementation of content-centric services in a heterogeneous vehicular environment was also presented to theoretically support the implementation of the heterogeneous vehicular network.

In the paper titled “Overcoming Challenges of Applying Reinforcement Learning for Intelligent Vehicle Control” [8], the authors apply Reinforcement Learning (RL) in intelligent vehicle control. They analyze the implications of RL in path-planning tasks. Concretely, first of all, they discuss the role of Curriculum Learning (CL) in structuring the learning process of intelligent vehicle control, not showing the learning examples randomly but organized instead in a meaningful order that gradually illustrates more concepts and gradually more complex ones. Second, they study a method to transfer RL policies from simulation to reality to make the agent experience situations in simulation so that it knows how to react to them in reality. To achieve this, they used 2D discrete grid environments with four possible states for each cell in the environment. Additionally, in the second set of experiments, they used the traffic junction environment as the learning environment. Finally, for the physical platform, they assembled multiple robots controlled by the Arduino Yún microcontroller. From the result of their experiments, they concluded the following: as the complexity of the environment influences the learning time and the performance of the agents when using intelligent vehicle control tasks such as path planning through reinforcement learning, several problems can arise, such as the existence of many possible states that the agent could experience, the existence of multiple agents, difficulty in representing states, or how we should formulate these safety-critical tasks to be solved by trial-and-error.

In [9] “On–Off Scheduling for Electric Vehicle Charging in Two-Links Charging Stations Using Binary Optimization Approaches”, the authors dealt with the problem of scheduling charging periods of electrical vehicles (EVs) to satisfy users’ demands for energy consumption as well as to optimally utilize available power. They proposed a new model for on–off scheduling of EV charging, assuming that every three-phase charger is equipped with two ports that can be served alternately. The scheduler considers individual charging rates and maximal currents that supply the entire farm separately for each phase. For this model, the authors analyzed several algorithms, and they concluded that binary quadratic programming solved with successive linear approximation algorithms satisfied the most important criteria and constraints in all statistical tests performed. This algorithm ensured smooth charging profiles but had a relatively long delay and was not the fastest. For this reason, the authors suggested that the issue of the algorithmic approach is still open, and further research in this area will be performed in the future.

The authors of the article “Advanced Driver Assistance Systems (ADAS) Based on Machine Learning Techniques for the Detection and Transcription of Variable Message Signs on Roads” [10] aimed at avoiding distractions when drivers pay attention to traffic signs. The authors developed a prototype of a Variable Message Sign (VMS) reading system using machine learning techniques. The assistant consists of two parts: one that recognizes a signal on the street and another that extracts its text and transforms it into speech. They used a neural network to recognize the VMS in an image and indicate its location with a confidence percentage.

Finally, in [11] “A Bidirectional Versatile Buck–Boost Converter Driver for Electric Vehicle Applications”, the authors presented a novel dc-dc bidirectional buck–boost converter between a battery pack and an inverter to regulate the dc bus in an electric vehicle powertrain. The converter was based on the versatile buck–boost converter, which has shown excellent performance in different fuel cell systems operating in low-voltage and hard-switching applications. The theoretical analyses were validated using simulations and experimental tests performed on a 400-V 1.6-kW prototype. The authors concluded that:

- The current controller regulated the traction of the dc bus during motoring and regenerative brake conditions.
- The system presented zero steady errors and a fast-transient response in the start-up for dc bus voltage reference changes and under realistic conditions using an EV powertrain system emulation.
